# [Corrigendum] Induction of microRNA‑let‑7a inhibits lung adeno­carcinoma cell growth by regulating cyclin D1

**DOI:** 10.3892/or.2024.8721

**Published:** 2024-03-05

**Authors:** Wei Zhao, Jin-Xia Hu, Rui-Min Hao, Qian Zhang, Jun-Qi Guo, You-Jie Li, Ning Xie, Lu-Ying Liu, Ping-Yu Wang, Can Zhang, Shu-Yang Xie

Oncol Rep 40: 1843–1854, 2018; DOI: 10.3892/or.2018.6593

After the publication of the article, an interested reader drew to the authors' attention that, in the western blots shown in Fig. 5C and D, a pair of data panels were inadvertently duplicated comparing between panels (C) and (D); in addition, the cell migration data shown in Fig. 7F on p. 1852 were selected incorrectly.

The authors have examined their original data, and realize that these errors arose inadvertently as a consequence of their mishandling of their data. The revised versions of Figs. 5 and 7, featuring the corrected data for the caspase-8 experiment in Fig. 5C and alternative data for the cell migration assay experiments in Fig. 7F, are shown on the next two pages. The revised data shown for these Figures do not affect the overall conclusions reported in the paper. All the authors agree to the publication of this corrigendum, and are grateful to the Editor of *Oncology Reports* for allowing them the opportunity to publish this. Furthermore, the authors apologize to the readership for any inconvenience caused.

## Figures and Tables

**Figure 5.**
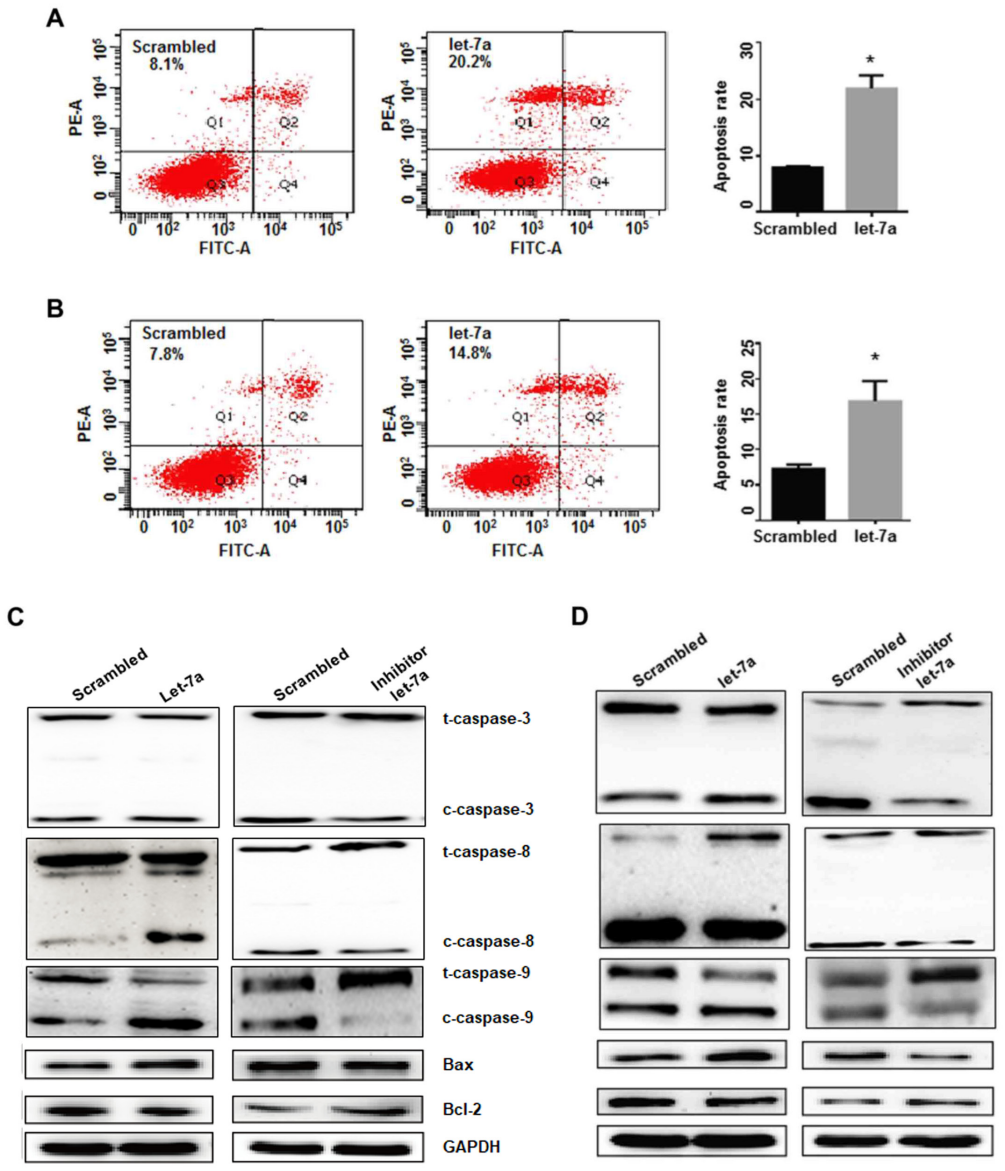
The effects of let-7a on cell apoptosis and gene expression. (A) Cell apoptosis in A549 cells was determined by flow cytometry. *P<0.05. (B) The effects of let-7a on apoptosis were analyzed in H1299 cells. *P<0.05. (C) Let-7a significantly downregulated Bcl-2 expression and upregulated the expression of Bax, cleaved-caspase-3, −8 and −9, compared with the scrambled control treatment in A549 cells. The let-7a-inhibitor increased the Bcl-2 levels and reduced Bax, and decreased cleaved-caspase-3, −8 and −9. (D) The expression of Bcl-2, Bax and cleaved-caspase-3, −8 and −9 was detected in the H1299 cell line. GAPDH was used as an internal control.

**Figure 7.**
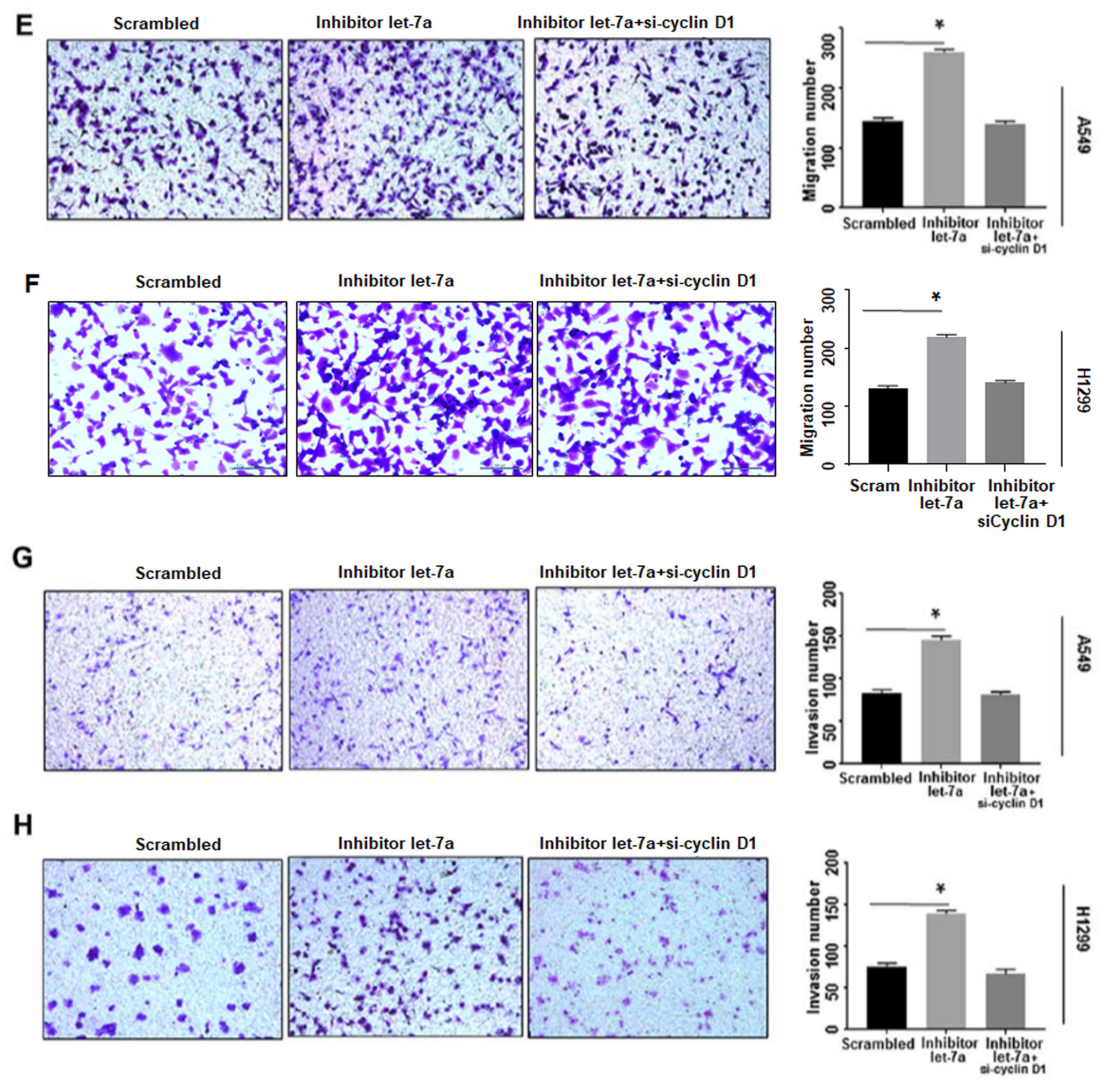
Continued. Let-7a suppresses cancer cell migration. (E and F) Let-7a inhibitor improved cell migration ability, which was reversed by cyclin D1-siRNA in A549 and H1299 cell lines. *P<0.01. (G and H) Cell invasion ability was improved by let-7a-inhibitor, which was reversed by cyclin D1-siRNA in A549 and H1299 cell lines. *P<0.01.

